# Oral Hemangiolymphangioma Presenting As Gingival Enlargement: A Rare Case With Literature Review

**DOI:** 10.7759/cureus.46674

**Published:** 2023-10-08

**Authors:** Supriya Jain, Sachin Dhingra, Shruti Tandon, Arundeep K Lamba, Farrukh Faraz

**Affiliations:** 1 Periodontics, Maulana Azad Institute of Dental Sciences, New Delhi, IND

**Keywords:** vascular anomalies, hemangiolymphangioma, gingival enlargement, gingiva, electrocautery

## Abstract

Vascular malformations are anomalies that are caused by disturbances in vasculogenesis. Depending upon the dominant structure present histologically, they may be found in different combinations of vascular elements and are named hemangiolymphangioma (HLA) or lymphangiohemangioma (LHA). HLA occurs in multiple anatomical sites, such as the head and neck, axilla, abdominal cavity, extremities, and urinary bladder, but it is infrequent in the oral cavity. An 18-year-old male with a history of abdominal tuberculosis presented with an asymptomatic mandibular gingival swelling that was histologically diagnosed as HLA. A six-month follow-up revealed no recurrence. The observations reported in this case are unusual, and our literature review revealed no previously documented case of gingival HLA.

## Introduction

Gingival enlargement is a predominant feature of periodontal disease, characterized by an increase in the size of the gingiva. Management depends on diagnosing the exact etiology. However, its presentation in various forms makes it difficult for the clinician to reach a specific diagnosis, especially when presented with unusual overgrowths in the oral cavity, such as vascular anomalies. 

Based on cellular turnover, histology, and clinical findings, vascular anomalies are categorized into two main types: vascular tumors and vascular malformation. Infantile hemangiomas constitute the majority of vascular anomalies and are contemplated as the predominant form of vascular tumors, composed of rapidly proliferating endothelial cells [1]. The latter include lymphangiomas, also known as lymphatic malformations, which are endothelial-lined cystic spaces formed by congenital collections of ectatic lymph vessels [2]. While malformations are structural abnormalities of blood vessels that exist since birth and do not regress with age, hemangiomas tend to develop in late fetal or early neonatal life and regress before or during puberty [3].

Lymphangioma seldom occurs in association with hemangioma. This mixed vascular anomaly is termed hemangiolymphangioma (HLA) or lymphangiohemangioma (LHA) according to the dominant component present microscopically. The literature states that the birth detection rates of HLA range from 40% to 60%, peaking at 80% to 90% during the first two years of life and declining with age [4]. HLA is seen predominantly in premature babies, and an incidence of 1:12000 is noticed in live newborns [4]. It can manifest in various anatomical regions, including the head and neck, axilla, abdominal cavity, extremities, pancreas, and urinary bladder. Only a few cases of HLA have been reported in the oral cavity, specifically the tongue, buccal mucosa, floor of the mouth, and mandible. Herein, we discuss an unusual case report of HLA, which presented clinically as gingival enlargement.

## Case presentation

An 18-year-old male reported in November 2022 to the Department of Periodontics, Maulana Azad Institute of Dental Sciences, with a chief complaint of swelling in the lower anterior tooth region that he first noticed in December 2021 due to bleeding while having food. During anamnesis, he mentioned that gingival swelling was sudden in onset with no change in size over time, but he experienced difficulty while eating solid food. The patient denied any history of trauma. He also mentioned that the appearance of the swelling was concomitant with his diagnosis of abdominal tuberculosis for which he completed anti-tubercular treatment (ATT) in October 2022. Family history was not relevant.

**Figure 1:**
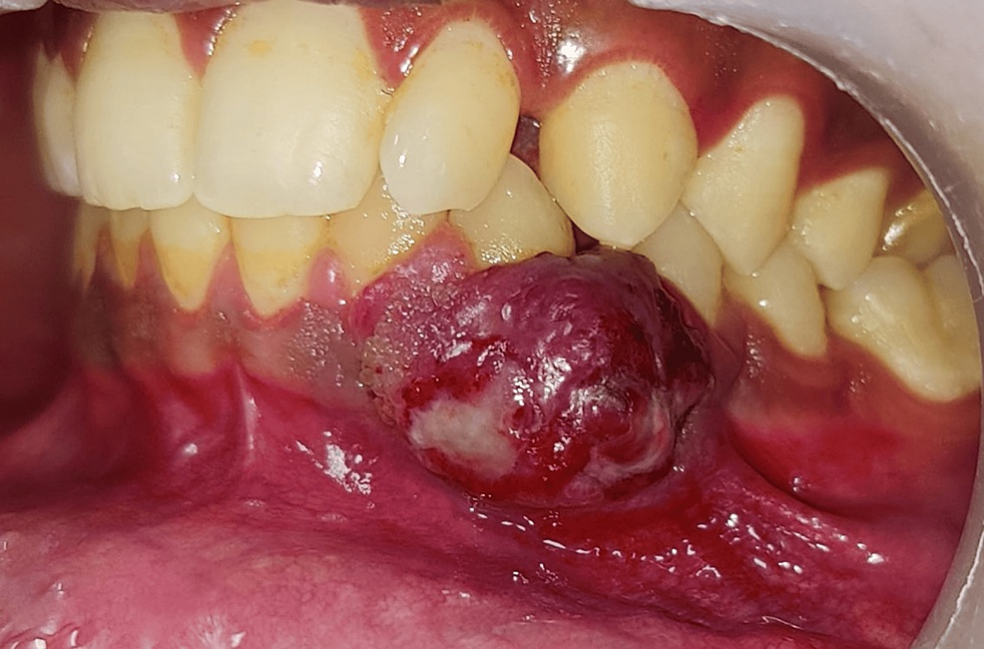
Preoperative view Preoperative frontal view showing an ovoid reddish-blue gingival growth in the lower anterior region

Extraoral examination revealed no relevant findings. On intraoral inspection, a sessile, well-defined ovoid, reddish-blue gingival growth with translucent yellow ulcerated areas and an irregular surface measuring approximately 1.2 x 1.5 cm was present on the labial aspect extending from the distal of 32 to the distal of 34, involving marginal gingiva, attached gingiva, and alveolar mucosa (Figure 1).

**Figure 2:**
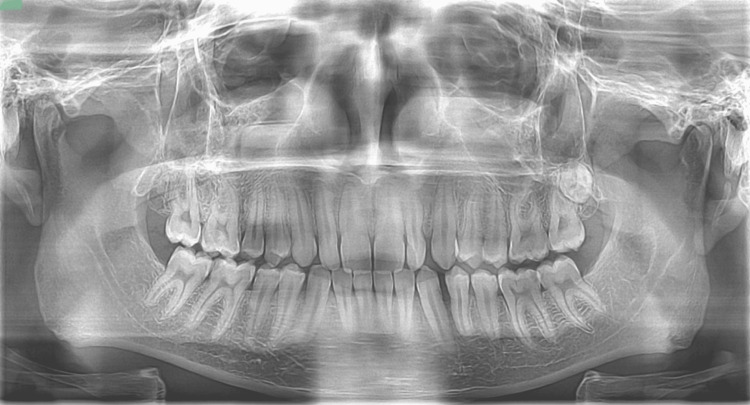
Radiographic view The radiographic picture reveals fine trabeculae of the bone present in the interdental region between 33 and 34.

On palpation, growth was non-tender, soft in consistency, fluctuant, compressible, but not reducible; no pulsatile thrill was felt and not fixed to the underlying structures. On auscultation, no bruit sound was heard. The radiographic picture revealed fine trabeculae of the bone in the interdental region between 33 and 34, resembling a cuffing bone resorption (Figure 2).

Hematological and biochemical parameters were within the normal range. Initially, oral prophylaxis was done, and oral hygiene instructions were given. Based on the appearance of the lesion and medical history, a provisional diagnosis of gingival tuberculosis was made.

**Figure 3:**
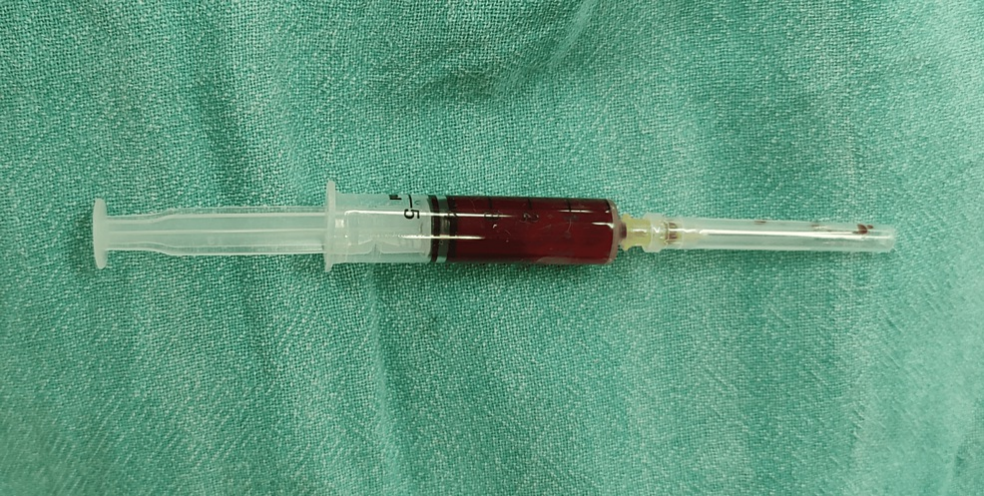
Fine-needle aspiration cytology (FNAC) sample

Fine-needle aspiration cytology (FNAC) done to rule out tuberculosis showed blood-tinted fluid that could not be used for the cartridge-based nucleic acid amplification test (CBNAAT) (Figure 3).

**Figure 4:**
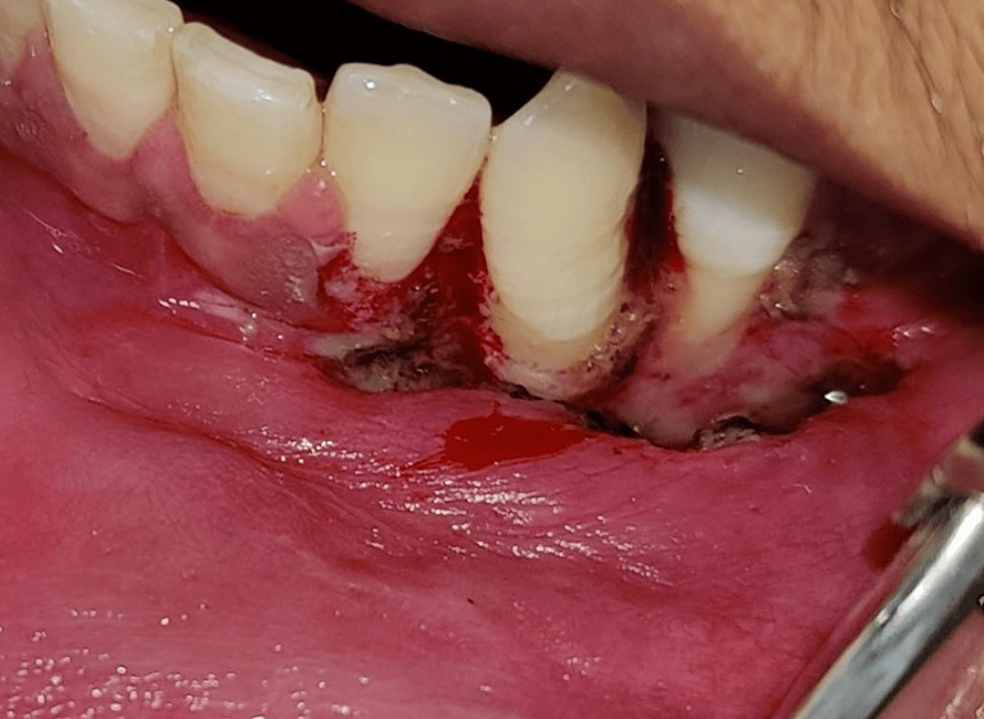
Immediate postoperative view

There was a spurt of blood after withdrawing the needle, which was managed by a pressure pack and cauterization of the area. After obtaining written informed consent from the patient, an excisional biopsy was planned on the same day under local anesthesia, 2% lignocaine with adrenaline (1: 80000), and aseptic conditions. Electrocoagulation was used for the excision. Primary closure could not be achieved and was left for healing by secondary intention, and a periodontal dressing was placed for seven days (Figure 4).

**Figure 5:**
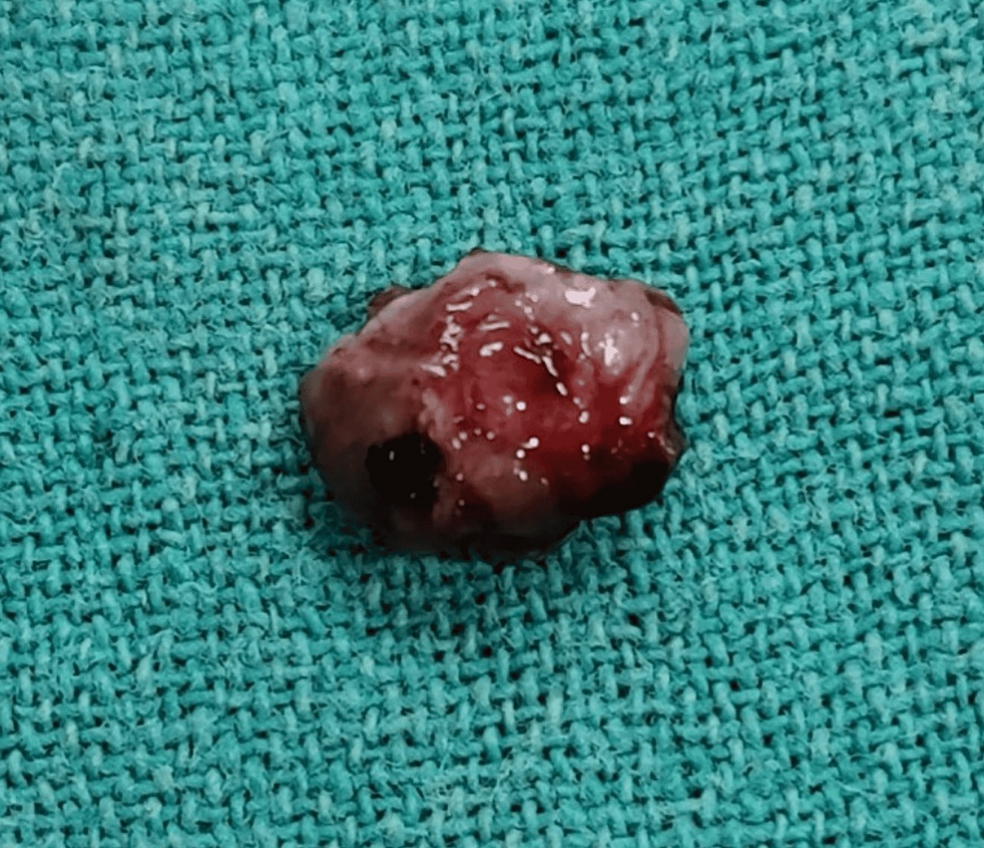
Gross biopsied specimen

The gross specimen, measuring roughly 1.2 x 0.9 cm, was sent for histopathological examination (Figure 5).

**Figure 6:**
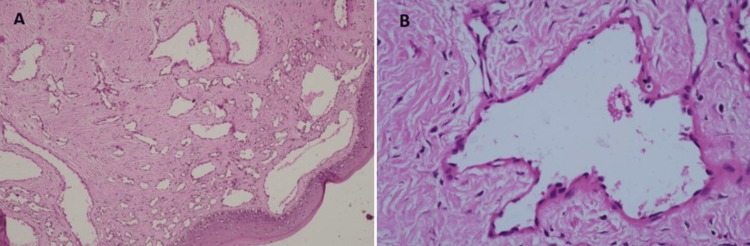
Photomicrographs of hematoxylin and eosin staining (A) Photomicrograph of hemangiolymphangioma at low magnification (10x) exhibiting large lymphatic vessels and numerous small-sized blood vessels. (B) Photomicrograph of hemangiolymphangioma at 40x magnification showing large lymphatic vessels lined by a single layer of flattened endothelial cells.

On microscopic examination, sections revealed para-keratinized stratified squamous epithelium beneath which lesional tissue was composed of large lymphatic vessels lined by a single layer of flattened endothelial cells. A few lymphatic channels were abutting the overlying epithelium. The connective tissue underneath this epithelium was densely collagenous, comprising collagen fibers arranged in a fascicular manner and spindle-shaped fibroblasts. At the other end, connective tissues showed numerous small-sized blood vessels lined by proliferating endothelial cells, intervened by collagen bundles with plump fibroblasts. This end was devoid of overlying epithelium (Figures 6A, 6B).

**Figure 7:**
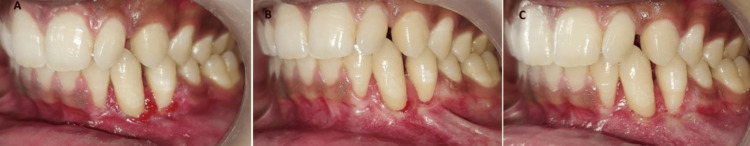
Postoperative view (A) 15-day follow-up, (B) three-month follow-up, (C) six-month follow-up

After 15 days, a postoperative evaluation was performed, and healing was uneventful. A follow-up of six months revealed no recurrence (Figures 7A-7C).

## Discussion

Twenty-two patients with HLA of the oral cavity have been documented, including the current case. A summary of previously reported cases was tabulated to review the literature (Table 1). 

**Table 1:** Reported cases of oral hemangiolymphangioma

Authors	Year	Age/ Sex	Site	Diagnosis	Recurrence
Vilalta et al. [5]	1985	7 y/M	Dorsum of the tongue	Hemangiolymphangioma	Not reported
Seong Soo et al. [6]	2003	62 y/M	Body mandible	Hemangiolymphangioma	Not reported
Jian et al. [7]	2005	2 y/M	Tongue	Lymphangiohemangioma	Not reported
Jian et al. [7]	2005	3 y/M	Tongue	Lymphangiohemangioma	Not reported
Jian et al. [7]	2005	4 y/M	Tongue	Lymphangiohemangioma	Not reported
Shetty D et al. [2]	2009	5 y/F	Dorsum of the tongue	Hemangiolymphangioma	Not reported
Shetty DC et al. [1]	2010	9 y/F	Dorsum of the tongue	Hemangiolymphangioma	Not reported
Sobhana et al. [8]	2012	18 y/M	Mucosa of the lip	Hemangiolymphangioma	Not reported
Duque et al. [9]	2013	6 y/M	Neck, floor of the mouth, tongue	Hemangiolymphangioma	Not reported
Hunchaisri [10]	2013	41 y/M	Floor of the mouth	Hemangiolymphangioma	Not reported
Yarmand et al. [11]	2016	26 y/M	Buccal mucosa	Lymphangiohemangioma	Not reported
Merhi et al. [12]	2016	2 y/M	Dorsum of the tongue	Hemangiolymphangioma	Not reported
De-Menezes et al. [13]	2017	6 y/F	Upper lip	Hemangiolymphangioma	Not reported
Manickam et al. [3]	2017	21 y/F	Buccal mucosa	Hemangiolymphangioma	Not reported
Reibero et al. [14]	2018	44 y/M	Dorsum of the tongue	Hemangiolymphangioma	Not reported
Deliverska [15]	2019	46 y/F	Mandible	Hemangiolymphangioma	Not reported
Khaunte et al. [4]	2020	5 y/M	Buccal mucosa	Hemangiolymphangioma	Not reported
De Souza Freitas et al. [16]	2020	25 y/M	Submandibular region	Hemangiolymphangioma	Not reported
De Azevedo et al. [17]	2020	7 y/F	Dorsum of the tongue	Hemangiolymphangioma	Not reported
de Souza Junior et al. [18]	2022	36 y/M	Lateral edge of the tongue	Venolymphatic malformation	Not reported
Ferreira-Santos et al. [19]	2023	23 y/M	Lateral edge of the tongue	Hemangiolymphangioma	Not reported

HLA in the oral cavity is rare, with only case reports in the literature. Based on the cumulative data, in the majority of patients, HLA was present on the tongue (12 out of 22), followed by the buccal mucosa (three out of 22 cases). The mean age occurrence of HLA is 18.9 years, with age ranging between two to 62 years, and it is most common in male patients, with sex distribution of male to female of 2.6:1 in the reviewed literature. To the best of our knowledge, this is the first case of HLA presenting as a gingival overgrowth. Although HLA is typically a benign disease, the invasion of underlying tissues and recurrence have been reported in the medical literature [20]. However, no evidence of recurrence has been reported in the reviewed cases by the authors.

Although the precise pathogenesis of HLA is unknown, theories have been put forth to explain its origin. The first theory states that during embryogenesis, the development of the primitive lymph channels is blocked or arrested, and the second theory states that the primitive lymphatic sac does not reach the vascular system [12].

The clinical characteristics of gingival HLA imitate pyogenic granuloma, chronic inflammatory gingival hyperplasia (epulis), epulis granulomatosa, and even squamous cell carcinoma and are critical for differential diagnosis. Moreover, gingival tuberculosis was suggested as a potential differential diagnosis in this case, based on the patient's medical history of tuberculosis and the clinical features of the growth. However, the histopathological characteristics of the specimen are what led to the final diagnosis. The biopsied samples in our case were all consistent with the histopathologic features of HLA, which displayed numerous dilated lymphatic channels along with multiple blood vessels.

The mainstay treatment for any vascular anomaly to lower the risk of recurrence is the excision of the lesion. Intralesional injection of sclerosing agents, interferon alpha-2 beta radiation, electrocoagulation, cryosurgery, embolization, and plasma knife are a few other treatment modalities that have been recommended in the literature for areas where lesions are not amenable to surgery (due to access, anatomic location, or proximity to vital structures) [4]. In the above case, treatment was done using electrocoagulation, and no recurrence was reported in the follow-up period.

## Conclusions

After a thorough review of the literature, gingival HLA has been found to be an uncommonly encountered tumor in the oral cavity. This case report emphasizes the importance of considering HLA as a differential diagnosis of gingival enlargement. Whenever a vascular anomaly is suspected, it is suggested that preoperative imaging, such as angiotomography, magnetic resonance angiography, and ultrasonography, should be advised to identify the feeder vessel, which can aid in the diagnosis and planning of the surgical procedure. Long-term follow-ups are recommended to rule out any recurrence or infiltration of the adjacent structures.

## References

[1] Shetty DC, Urs AB, Rai HC, Ahuja N, Manchanda A (2010). Case series on vascular malformation and their review with regard to terminology and categorization. Contemp Clin Dent.

[2] Shetty D, Rai H, Rastogi P, Panda A, Ahuja N (2009). Vascular malformations of the oral cavity in children and young adolescents - insights into their pathogenesis. Internet J Pediat Neonatol.

[3] Manickam S, Sasikumar P, Kishore BN, Joy S (2017). Hemangiolymphangioma of buccal mucosa: a rare case report. J Oral Maxillofac Pathol.

[4] Khaunte DDN, Kumar PS, Dhupar V, Naik M (2020). Hemangiolymphangioma of buccal cheek- a rare case report with review of literature. J Dent Health Oral Disord Ther.

[5] Vilalta J, Mascaro JM (1985). Hemangiolymphangioma of the tongue treated by transfixion technique. J Dermatol Surg Oncol.

[6] (2022). Intraosseous haemangiolymphangioma of the mandible: a case report. https://www.jkaoms.org/journal/view.html.

[7] Jian XC (2005). Surgical management of lymphangiomatous or lymphangiohemangiomatous macroglossia. J Oral Maxillofac Surg.

[8] Sobhana CR, Beena VT, Soni A, Choudhary K, Sapru D (2012). Hemangiolymphangioama of buccal mucosa: report of a rare case and review of literature on treatment aspect. Natl J Maxillofac Surg.

[9] Duque CS, Londoño AF, Penagos AM, Urquijo DP, Dueñas JP (2013). Hypoglossal nerve monitoring, a potential application of intraoperative nerve monitoring in head and neck surgery. World J Surg Oncol.

[10] (2022). Hemangiolymphangioma of the floor of mouth: a case report and literature review. https://ejournals.swu.ac.th/index.php/JMHS/article/view/4222/0..

[11] Yarmand F, Seyyedmajidi M, Shirzadc A, Foroughi R, Bakhshian A (2016). Lymphangiohemangioma of buccal mucosa: report of a rare case. J Oral Maxillofac Surg Med Pathol.

[12] (2022). Hemangiolymphangioma of the tongue, report of a rare case. https://www.researchgate.net/publication/344630558_HemangioLymphangioma_of_Tongue_Report_of_a_Rare_Case..

[13] De-Menezes RER, Gouvêa AF, Takahama A, Lopes DN, Da Costa Fontes KBF, De Souza Azevedo R, Boziki A (2017). A rare case of oral haemangioma. Oral Surg Oral Med Oral Pathol Oral Radiol.

[14] Ribeiro GA, Dias AMR, Leite AFSA, Buexm LA, Lima GS, Alves ATNN, Lourenco SDQC (2018). Oral hemangiolymphangioma: a case report. Oral Surg Oral Med Oral Pathol Oral Radiol.

[15] Deliverska E (2019). Hemangiolymphangioma of the mandible: case report. J Int Med Assoc Bulg.

[16] De Souza Freitas E, Da Cunha RG, Leite MGM, De Lima Souza Y, Alves TM, Ferreira FAB, Câmara J (2020). Hemangiolymphangioma in submandibular region: a case report. Oral Surg Oral Med Oral Pathol Oral Radiol.

[17] De Azevedo AB, Satake HH, Faustino ISP, Curioso PAB, De Almeida OP, Silva AS, Lopes MA (2020). Hemangiolymphangioma: a case report. Oral Surg Oral Med Oral Pathol Oral Radiol.

[18] de Souza EF, Sena DA, Lucena VR, de Souza LB, de Morais HH (2022). Venolymphatic malformation in lateral edge of the tongue: case report. J Vasc Bras.

[19] Ferreira-Santos RI, Santos KA, Scherma AP, León JE, Kaminagakura E (2023). Unveiling an oral hemangiolymphangioma. Autops Case Rep.

[20] Mao CP, Jin YF, Yang QX, Zhang QJ, Li XH (2018). Radiographic findings of hemolymphangioma in four patients: a case report. Oncol Lett.

